# Poleward-propagating near-inertial waves enabled by the western boundary current

**DOI:** 10.1038/s41598-019-46364-9

**Published:** 2019-07-09

**Authors:** Chanhyung Jeon, Jae-Hun Park, Hirohiko Nakamura, Ayako Nishina, Xiao-Hua Zhu, Dong Guk Kim, Hong Sik Min, Sok Kuh Kang, Hanna Na, Naoki Hirose

**Affiliations:** 1 Department of Marine Science and Biological Engineering, Inha University, Incheon, Korea; 2 Department of Ocean Sciences, Inha University, Incheon, Korea; 3 Faculty of Fisheries, Kagoshima University, Kagoshima, Japan; 4 State Key Laboratory of Satellite Ocean Environment Dynamics, Second Institute of Oceanography, Ministry of Natural Resources, Hangzhou, China; 5 Southern Marine Science and Engineering Guangdong Laboratory (Zhuhai), Zhuhai, China; 6 Korea Institute of Ocean Science and Technology, Busan, Korea; 7 Seoul National University, Seoul, Korea; 8 Research Institute for Applied Mechanics, Kyushu University, Kasuga, Japan

**Keywords:** Physical oceanography, Physical oceanography

## Abstract

Near-inertial waves (NIWs), which have clockwise (anticlockwise) rotational motion in the Northern (Southern) Hemisphere, exist everywhere in the ocean except at the equator; their frequencies are largely determined by the local inertial frequency, *f*. It is thought that they supply about 25% of the energy for global ocean mixing through turbulence resulting from their strong current shear and breaking; this contributes mainly to upper-ocean mixing which is related to air-sea interaction, typhoon genesis, marine ecosystem, carbon cycle, and climate change. Observations and numerical simulations have shown that the low-mode NIWs can travel many hundreds of kilometres from a source region toward the equator because the lower inertial frequency at lower latitudes allows their free propagation. Here, using observations and a numerical simulation, we demonstrate poleward propagation of typhoon-induced NIWs by a western boundary current, the Kuroshio. Negative relative vorticity, meaning anticyclonic rotational tendency opposite to the Earth’s spin, existing along the right-hand side of the Kuroshio path, makes the local inertial frequency shift to a lower value, thereby trapping the waves. This negative vorticity region works like a waveguide for NIW propagation, and the strong Kuroshio current advects the waves poleward with a speed ~85% of the local current. This finding emphasizes that background currents such as the Kuroshio and the Gulf Stream play a significant role in redistribution of the NIW energy available for global ocean mixing.

## Introduction

Ocean mixing, mainly driven by wind and tides, is known to play a crucial role in maintaining oceanic stratification and the global-scale thermohaline circulation^1^. The ocean needs about 2.1 terawatt of energy for mixing, and NIWs are responsible for 0.4–0.7 terawatt^1–4^, approximately 25% of the total. NIWs exist everywhere in the ocean (except at the equator) from tropical to polar regions^2,5^. They redistribute energy through long-distance propagation in stratified waters and are dissipated eventually through turbulence due to their high shear^3^. Nevertheless, because of low spatial resolution (>1/4 degree) and low temporal resolution of the wind forcing (>6 hours), climate and Earth system models which are used for future climate prediction do not represent realistic NIWs and their mixing^6–8^.

NIWs travel over hundreds of kilometres toward the equator because of decreasing inertial frequency with latitude^2,3,9–11^. Negative relative vorticity induced by horizontal current shear can trap and amplify NIWs^10,12^ with enhancement of NIW energy transfer to the deep ocean^12–15^. Numerical simulation and observations have shown that the Gulf Stream and Kuroshio, the western boundary currents in the Atlantic and Pacific Oceans, can move the waves eastward away from their sources, and hence produce a NIW “desert” south of a western boundary current^16,17^. Nevertheless, our understanding of the behaviour of non-equatorward NIW propagation is insufficient, largely due to lack of observations, especially in strong current regions.

**Figure 1:**
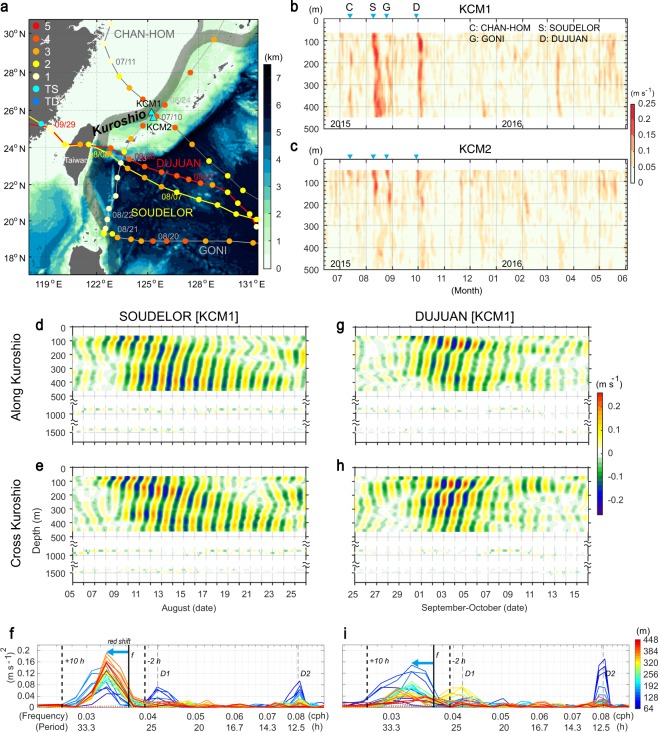
Typhoon tracks and NIWs. (**a**) Typhoon tracks and Saffir-Simpson scale (lines, and dots every 6 hours) during the observation period, 06/2015–05/2016. Dots with mm/dd date designations mark typhoon positions at 00 h GMT on those dates. Background colours are bathymetry (km). Black shaded area is the typical Kuroshio path. Two mooring sites, KCM1 and KCM2, are marked with cyan triangles. The most energetic NIWs observed were generated by the passages of two typhoons, SOUDELOR (yellow) and DUJUAN (red). (**b,c**) NIW amplitude at KCM1 and KCM2 from upward-looking ADCPs moored at about 500-m depth. Inverted cyan triangles indicate the times when the typhoons (each designated by its initial letter) passed across the Kuroshio core. Tick marks on the time axes correspond to the beginnings (00 h GMT on Day 1) of the designated months. (**d–f**) Time series of along-Kuroshio and cross-Kuroshio directional NIW velocities from an upward-looking 75-kHz ADCP and current metres (1000-m and 1500-m depths) at KCM1 for the typhoon SOUDELOR. Tick marks on the time axes correspond to the beginnings (00 h GMT) of the designated days. Variance-preserving rotary power spectra during the passage of SOUDELOR. (**g–i**) Same but for DUJUAN. In (**f,i**) Solid lines are clockwise components and dotted lines (faint and weak) are anticlockwise components. Vertical solid line is the local inertial frequency (*f*) and vertical dashed lines indicate the near-inertial frequency band for analysis. Vertical dotted lines mark the diurnal (D1) and semidiurnal (D2) frequencies. Colours indicate current-measurement depths.

Wind is the best-known forcing capable of generating energetic NIWs in the ocean. A typhoon or hurricane generates energetic NIWs, especially on the right-hand side of its track because of the stronger wind energy^18^ and more favourable wind-direction change. The Joint Kuroshio-Ryukyu Current System Study (JKRYCSS), an international collaborative project to observe the Kuroshio and Ryukyu Current systems, began in June 2015, and tall current moorings were installed in the Okinawa Trough to monitor the Kuroshio where it flows northeastward. While the current moorings were in operation, four typhoons passed over or near the two mooring sites: CHAN-HOM in July, SOUDELOR in early August, GONI in late August, and DUJUAN in late September (Fig. 1a). Time series of current profiles from upward-looking 75-kHz acoustic Doppler current profilers (ADCP) reveal particularly energetic NIWs after the passage of SOUDELOR and of DUJUAN, which passed across a region of the Kuroshio upstream from the mooring sites (Fig. 1b,c). Amplitudes of the waves induced by the typhoons were stronger than those during the winter season. When passing through the region near the mooring sites the four typhoons had similar intensities, category 4 for CHAN-HOM, GONI, and DUJUAN and category 3 for SOUDELOR. Typhoons CHAN-HOM and GONI passed closest to the mooring sites, but the other two typhoons, SOUDELOR and DUJUAN, produced stronger NIW signals at the KCM1 and KCM2 mooring sites, particularly at KCM1 (Fig. 1b,c).

The strongest observed NIW events were responses to the two typhoons SOUDELOR and DUJUAN, which crossed the Kuroshio upstream, east of Taiwan, about 350 km southwest of the mooring sites on August 8^th^ and September 28^th^, respectively (Fig. 1a); these events lasted 15 and 9 days, respectively. In both cases, the wave amplitude was stronger at KCM1 than at KCM2 (Fig. 1b,c). Energetic NIWs at KCM1 for SOUDELOR were observed from August 8^th^ at 64 m to August 23^rd^ at 448 m, exhibiting downward propagation (Fig. 1d,e). For DUJUAN, energetic NIWs were detected from September 29^th^ at 64 m to October 7^th^ at 448 m, again exhibiting downward propagation (Fig. 1g,h). The waves at about 1000- and 1500-m depths had amplitudes less than 0.04 m s^–1^, which is much weaker than in the upper layer. The clockwise component of the NIWs (solid lines in Fig. 1f,i) was stronger than the anticlockwise component (dotted lines), which is an obvious characteristic of NIWs in the Northern Hemisphere. Frequencies of the NIWs are smaller than the local inertial frequency (*f*) at all measurement depths, 64–448 m, during both typhoon passages (Fig. 1f,i). The local inertial period is 27.47 hours (*f* = 0.0364 cph) at KCM 1, but, with this so-called red-shift, the period of NIWs determined from the spectral analysis was 30.3 hours (=0.033 cph) (see Methods), corresponding to ~0.9 *f*.

**Figure 2:**
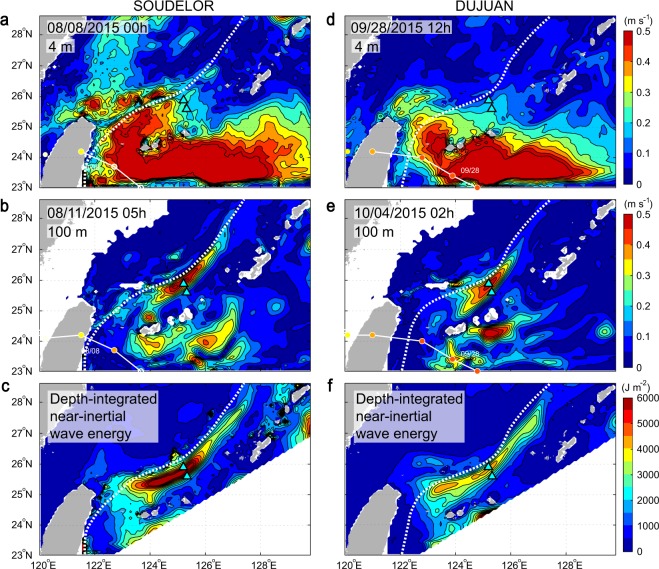
NIW amplitude and kinetic energy for passages of two typhoons, SOUDELOR and DUJUAN, calculated from real-time ocean forecasting model data. (**a**) Snapshot of NIW amplitude near the sea surface (4-m depth) for typhoon SOUDELOR on August 8^th^, 2015 (00 h GMT). (**b**) Snapshot of NIW amplitude at 100-m depth on August 11^th^ (05 h GMT) for SOUDELOR. (**c**) Depth-integrated NIW kinetic energy over 20 days during typhoon SOUDELOR. (**d**) Snapshot of NIW amplitude near the sea surface (4-m depth) for typhoon DUJUAN on September 28^th^, 2015 (12 h GMT). (**e**) Snapshot of NIW amplitude at 100-m depth on October 4^th^ (02 h GMT) for DUJUAN. (**f**) Depth-integrated NIW kinetic energy over 20 days during typhoon DUJUAN. Triangles in Fig. 2 are observation stations, KCM1 and KCM2, as shown in Fig. 1a.

Comparisons of NIWs from observations and numerical simulations reveal good agreements in both amplitude and frequency-shift, and the characteristic that the NIWs were stronger at KCM1 than at KCM2 in the observations is well represented in the simulations (Supplementary Figs S1–3). Numerical simulation results (see Methods) show typhoon-SOUDELOR-induced energetic NIWs distributed widely near the sea surface (4-m depth) in the Kuroshio upstream about 100 km southwest from the mooring sites. The simulated waves are weak (<0.25 m s^−1^) at the mooring sites at 00 h GMT on August 8^th^ when the most intense NIWs are generated by typhoon SOUDELOR, and the waves in the Kuroshio downstream and on the northern side of the mooring sites are weaker still (Fig. 2a, Supplementary Video 1). The waves at 100-m depth become energetic ~3 days later along the Kuroshio downstream (over about 300 km including the mooring sites), suggesting poleward energy propagation (Fig. 2b, Supplementary Video 2). Depth-integrated kinetic energy greater than 3000 J m^−2^ exists along the right-hand side of the Kuroshio path from 25°N to 27.6°N (Fig. 2c), resulting from energy propagation downstream along the Kuroshio. During typhoon DUJUAN, generation of NIWs in the surface layer (4 m) is weak (<0.2 m s^−1^) at the mooring sites, while strong (>0.4 m s^−1^) in the Kuroshio upstream, roughly 2 times farther from observation sites than during typhoon SOUDELOR (Fig. 2d, Supplementary Video 3). After ~5.5 days, prominent NIWs exist at and around mooring sites along the Kuroshio at 100-m depth (Fig. 2e, Supplementary Video 4). Depth-integrated kinetic energy shows the same spatial distribution along the right-hand side of the Kuroshio path in the case of DUJUAN from about 25°N to 28°N (Fig. 2f), similar to that of typhoon SOUDELOR. The NIWs were stronger at KCM1 than at KCM2 from both observations and simulations due to the location of KCM1 which is close to the center of the right-hand side of the Kuroshio, where the NIW energy was more concentrated.

**Figure 3:**
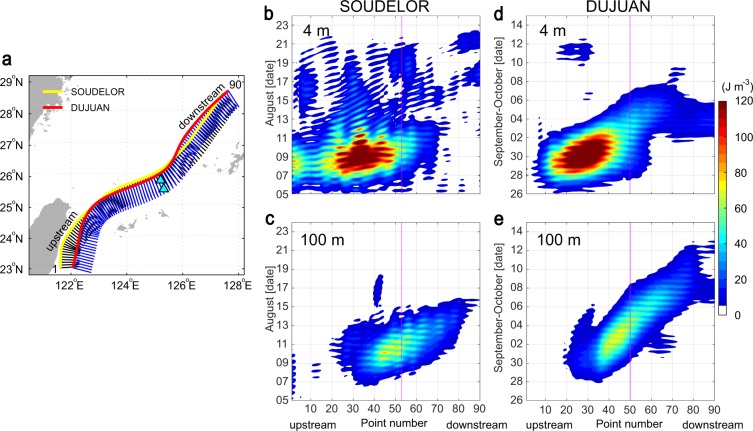
Kuroshio paths and Hovmöller diagram of NIWs along the right-hand side of the Kuroshio path averaged over 80-km range, during typhoons SOUDELOR and DUJUAN. (**a**) Kuroshio paths and their right-hand-side perpendicular lines (0–80 km) for typhoons SOUDELOR and DUJUAN. (**b,c**) Hovmöller diagram of NIW kinetic energy density averaged over 80 km to the right of the axis, near the sea surface (4-m depth) in (**b**) and 100 m in (**c**) for typhoon SOUDELOR. (**d,e**) As for (**b,c**) respectively, but for typhoon DUJUAN. Magenta-coloured vertical line indicates the perpendicular line corresponding to the observation locations.

The Kuroshio path and its perpendicular lines (Fig. 3a; see Methods) and Hovmöller diagrams of NIW kinetic energy density averaged over the right-hand side of the Kuroshio from the centre to 80 km away from the centre during typhoons SOUDELOR and DUJUAN (Fig. 3b–e), show the detailed behaviour of NIWs along that side of the Kuroshio (Supplementary Videos 5 and 6). The NIW kinetic energy density at the sea surface are weak (<40 J m^−3^) around the mooring sites when SOUDELOR and DUJUAN go through, however they become strong 1–2 days and 2–3 days later at the sea surface (Fig. 3b,d) and ~3 days and 5.5 days later at 100-m depth (Fig. 3c,e), respectively. The kinetic energy density at the sea surface shows energy movement from upstream to downstream with speed of 1.5 m s^−1^ for SOUDELOR and 0.93 m s^−1^ for DUJUAN on average, almost the same speed of the Kuroshio near the axis. The advection of NIWs at the sea surface shows similar patterns (Supplementary Videos 1 and 3) as that in the Gulf Stream^17^. The energy movement at 100-m depth exhibits 0.58 m s^−1^ for SOUDELOR and 0.43 m s^−1^ for DUJUAN on average, about 85% of the Kuroshio speed on its right-hand side for both cases.

**Figure 4:**
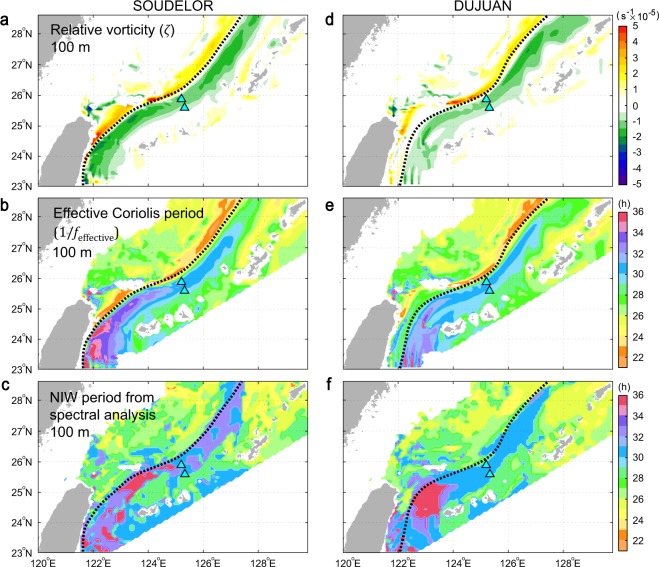
Relative vorticity, effective Coriolis period, and NIW period from spectral analysis calculated from the real-time ocean forecasting model data. (**a,b**) Relative vorticity and effective Coriolis period at 100-m depth during typhoon SOUDELOR (20-day average). (**c**) NIW period from spectral analysis of 100-m currents for the 20-day SOUDELOR period. (**d–f**) As for (**a–c**) respectively, but for typhoon DUJUAN.

Numerical simulation results reveal a negative relative vorticity (see Methods) region with 80–90 km width on the right-hand side of the Kuroshio path during the passage of SOUDELOR and of DUJUAN (Fig. 4a,d). Due to this negative relative vorticity, the effective inertial period 1/*f*_*eff*_, where *f*_*eff*_ is effective Coriolis frequency (see Methods), is longer than 30 hours (approximately 10% longer than the local inertial period 1/*f*) along the right-hand side of the Kuroshio up to about 27.5°N (Fig. 4b,e). This implies that NIWs generated in the upstream region of the Kuroshio can be trapped and their energy propagated in a band along the right-hand side of the Kuroshio. This is supported by the fact that NIWs in this band reveal periods similar to 1/*f*_*eff*_ (Fig. 4c,f). In other words, the negative relative vorticity region works like a waveguide for NIWs. Moreover, the Kuroshio itself advects these NIWs northeastward (poleward) more than 300 km.

The Hovmöller diagram of NIWs along the right-hand side of the Kuroshio shows slight upstream directional phase propagation, the speed of which is faster than 4.5 m s^−1^ (Fig. 3b–e). This unrealistic propagation speed can be explained with spatially different phase of NIWs along the Kuroshio. The Hovmöller diagram of NIWs shows temporally fixed NIWs along the Kuroshio at the sea surface and 100-m depth over hundreds of kilometres. Because the NIWs move downstream over time with a speed of >50 km day^−1^ and with downward propagation of their energy with O(10) m day^−1^, the NIWs at 100-m depth can have different phase at a fixed time (Figs 1, S1). If the NIW phase in the downstream is earlier than in the upstream, there could be an upstream directional phase propagation. The spatially different NIW phase can also happen by different frequency of NIWs along the Kuroshio (Fig. 4c,f).

Typhoons CHAN-HOM and GONI passed close to the mooring sites (Fig. 1a), generating strong NIWs in the surface layer around the sites. Nevertheless, the observations show no distinguishable NIWs spreading from the 64 to 448 m depths at KCM1 since they are advected to downstream regions by the Kuroshio (Figs 1b,c, S4 and Supplementary Videos 7–10). This advection removes NIW energy from the source region in the upper layer, which accounts for the observed weak deep-layer NIWs, <0.04 m s^−1^, at 1000- and 1500-m depths (Fig. 1d,e,g,h). The energetic NIWs are found at KCM2 during typhoon GONI due to its position on the right-hand side of the typhoon track. The relatively slow Kuroshio current at KCM2 enables to sustain the locally generated NIWs.

Following generation, the distribution of NIWs is traditionally thought to be governed by equatorward propagation, though the potential impact of background currents on propagation has been noted without supporting evidence^3,5,10,19^. The observations and simulations presented in this study provide a new paradigm suggesting that NIWs can be advected northward over hundreds of kilometres along a western boundary current. NIWs can mix waters through turbulence by their current shear and breaking^2,3,6,15,20,21^, and as a result can influence pollutant dispersal, the marine ecosystem, carbon cycle, and climate^22^. Our results suggest that proper incorporation of NIW effects in climate and Earth system models will require consideration of the impact of background currents on wave energy redistribution, resulting in further improvements of future climate predictions.

## Data and Methods

### *In-situ* moored measurements

Two sets of current measuring mooring systems were deployed in the Kuroshio from June 2015 to June 2016. Each system consisted of an upward-looking 75-kHz Acoustic Doppler Current Profilers (ADCP) at about 500-m depth, and single-level-measuring current metres at 1000-m and 1500-m depths with sampling interval of 30 minutes. The uncertainty of the 30-minute-interval ADCP data in configuration of 16-m bin size and 10 pings ensemble from Workhorse Long Ranger 75-kHz ADCP (20° beam angle) is 1.23 cm s^−1^. The two stations, KCM1 and KCM2, were positioned on the right-hand side of the Kuroshio path, with KCM1 close to the Kuroshio path (core) and KCM2 near its edge. The two stations were separated by about 34 km distance (Fig. 1a). Ocean depths at KCM1 and KCM2 are 2004 m and 2051 m, respectively.

### Numerical model

Data-assimilative three-dimensional high-resolution numerical ocean model outputs (hourly) from the real-time forecast system covering the East Asian marginal seas were analyzed [https://dreams-c.riam.kyushu-u.ac.jp/vwp/]^23^. This system has horizontal resolutions of 1/12° in longitude and 1/15° in latitude, based on the RIAM Ocean Model (RIAMOM)^24^, a free-surface primitive general circulation model developed by the Research Institute for Applied Mechanics (RIAM) of Kyushu University. It is a three-dimensional, 38-layer, *z*-coordinate model that assumes hydrostatic balance and Boussinesq approximation. 32 of these 38 layers covers from the surface to 2250 m. The model includes tides^25^ and ocean general circulation and is forced by 6-hourly atmospheric forcings (GPV/GSM meteorological data) with 3rd-order Lagrange polynomial interpolation. Sea surface temperature data (MGDSST) of the Japan Meteorological Agency were utilized for the surface relaxation with a time scale of 3 days, and the along-track AVISO sea surface height data were assimilated by a reduced-order Kalman filter.

### Horizontal energy

Time-mean depth-integrated horizontal kinetic energy of NIWs was calculated by $HKE=\frac{1}{2T}{\int }_{-H}^{0}({\int }_{0}^{T}\rho (z,t)[\,{|{u}^{\text{'}}(z,t)|}^{2}+{|{v}^{\text{'}}(z,t)|}^{2}]dt)dz,$ where *t* is time, *z* is the vertical Cartesian coordinate (positive upward), *ρ* is water density, *T* ( = 20 days) is an averaging period, and ${u}^{\text{'}}$ and ${v}^{\text{'}}$ are baroclinic zonal and meridional velocities filtered to the near-inertial frequency band^26–28^. This filter was a third-order Butterworth phase-preserving band-pass filter applied with cutoffs at −2 and +10 hours from the local inertial period. These cutoff periods, based on rotary spectral analysis, were chosen to prevent diurnal tide (D1) interference and took into consideration frequency shifts of the NIWs (Fig. 1f,i). The variance-preserving rotary spectral analysis^29^, which provides clockwise and anticlockwise rotating components, was conducted with 20-day-long current data during each typhoon passage. The duration of 20 days was necessary to separate the diurnal tides and NIWs. The start date of the duration of 20 days for horizontal energy and spectral analysis is 00 h GMT on August 5^th^ and on September 26^th^ during SOUDELOR and DUJUAN, respectively.

### Kuroshio path and perpendicular lines

The Kuroshio path and its perpendicular lines were estimated. The Kuroshio path was determined as follows. First, the current field was 4-day lowpass filtered and the maximum surface velocity points were identified in these currents averaged over 20 days, corresponding to the duration of the spectral analysis. Points along the Kuroshio path were determined every 10-km and numbered 1 to 90 ranging from 23°N to about 28.5°N.

### Effective Coriolis frequency

The effective Coriolis frequency is given by ${f}_{eff}=f+\zeta /2$, where *ζ* is the relative vorticity, $\zeta =\partial v/\partial x-\partial u/\partial y$^10^. Negative (positive) *ζ* denotes anticyclonic (cyclonic) circulation.

## Supplementary information

Supplementary Video 1 SOUDELOR_NIW_Hori_4m

Supplementary Video 2 SOUDELOR_NIW_Hori_100m

Supplementary Video 3 DUJUAN_NIW_Hori_4m

Supplementary Video 4 DUJUAN_NIW_Hori_100m

Supplementary Video 5 SOUDELOR_Along_Kuro_100m

Supplementary Video 6 DUJUAN_Along_Kuro_100m

Supplementary Video 7 CHANHOM_NIW_Hori_4m

Supplementary Video 8 CHANHOM_NIW_Hori_100m

Supplementary Video 9 GONI_NIW_Hori_4m

Supplementary Video 10 GONI_NIW_Hori_100m

SUPPLEMENTARY INFO

## Data Availability

Typhoon tracks and Saffir-Simpson scale are available at http://www.metoc.navy.mil/jtwc/jtwc.html, *STRM30_PLUS* global topography data are from http://topex.ucsd.edu/WWW_humtl/strm30_plus.html, and the typical Kuroshio path is obtained from http://www.khoa.go.kr/koofs/kor/seawf/sea_wflow.do?menuNo=02&link. All other data are available from the corresponding author upon reasonable request.
