# Supplementary material for: Poleward-propagating near-inertial waves enabled by the western boundary current
Source: Sci Rep. 2019 Jul 9;9:9955. doi: 10.1038/s41598-019-46364-9 (PMC6616354; doi:10.1038/s41598-019-46364-9)
Supplement: Supplementary file 11 — SUPPLEMENTARY INFO [file 41598_2019_46364_MOESM11_ESM.docx]

<Supplementary information>

Poleward-propagating near-inertial waves enabled by the western boundary current

Chanhyung Jeon^1^, Jae-Hun Park^2^, Hirohiko Nakamura^3^, Ayako Nishina^3^, Xiao-Hua Zhu^4,5^, Dong Guk Kim^6^, Hong Sik Min^6^, Sok Kuh Kang^6^, Hanna Na^7^, Naoki Hirose^8^

^1^Department of Marine Science and Biological Engineering, Inha University, Incheon, Korea

^2^Department of Ocean Sciences, Inha University, Incheon, Korea

^3^Faculty of Fisheries, Kagoshima University, Kagoshima, Japan

^4^State Key Laboratory of Satellite Ocean Environment Dynamics, Second Institute of Oceanography, Ministry of Natural Resources, Hangzhou, China

^5^Southern Marine Science and Engineering Guangdong Laboratory (Zhuhai), Zhuhai, China

^6^Korea Institute of Ocean Science and Technology, Busan, Korea

^7^Seoul National University, Seoul, Korea

^8^Research Institute for Applied Mechanics, Kyushu University, Japan

Corresponding author: jaehunpark@inha.ac.kr

Comparing time series of observations and numerical simulations of NIWs at KCM1 (S. Fig. 1) and KCM2 (S. Fig. 2), we see good agreement between them in the cases of typhoon SOUDELOR and of typhoon DUJUAN. Variance-preserving rotary power spectra show red-shifted frequencies of NIWs in the model results and observations at both KCM1 and KCM2 (S. Fig. 3).


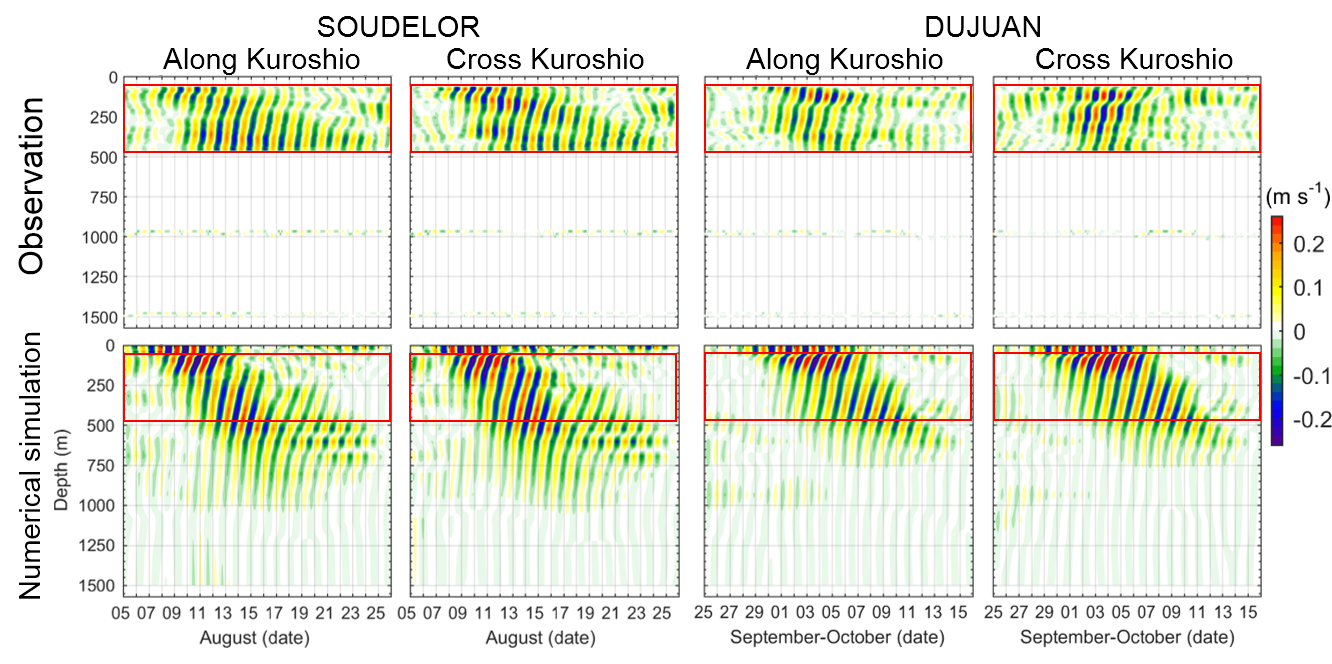


Figure S1 Comparison of time series of along-Kuroshio and cross-Kuroshio directional NIW velocities from observation and numerical simulation at KCM1 for typhoons SOUDELOR and DUJUAN. For convenience, the rectangular red box shows the region of upward-looking ADCP observations. Tick marks on the time axes correspond to the beginnings (00 h GMT) of the designated days.


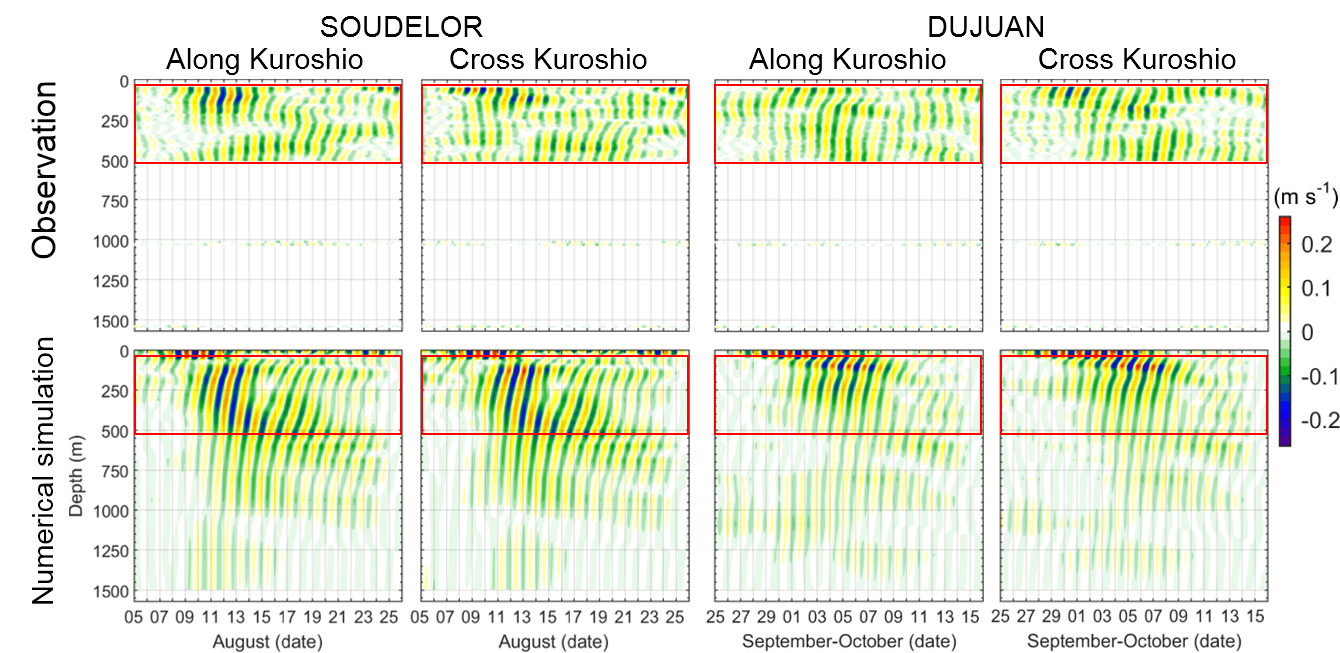


Figure S2 Same as S. Figure 1 but for KCM2.


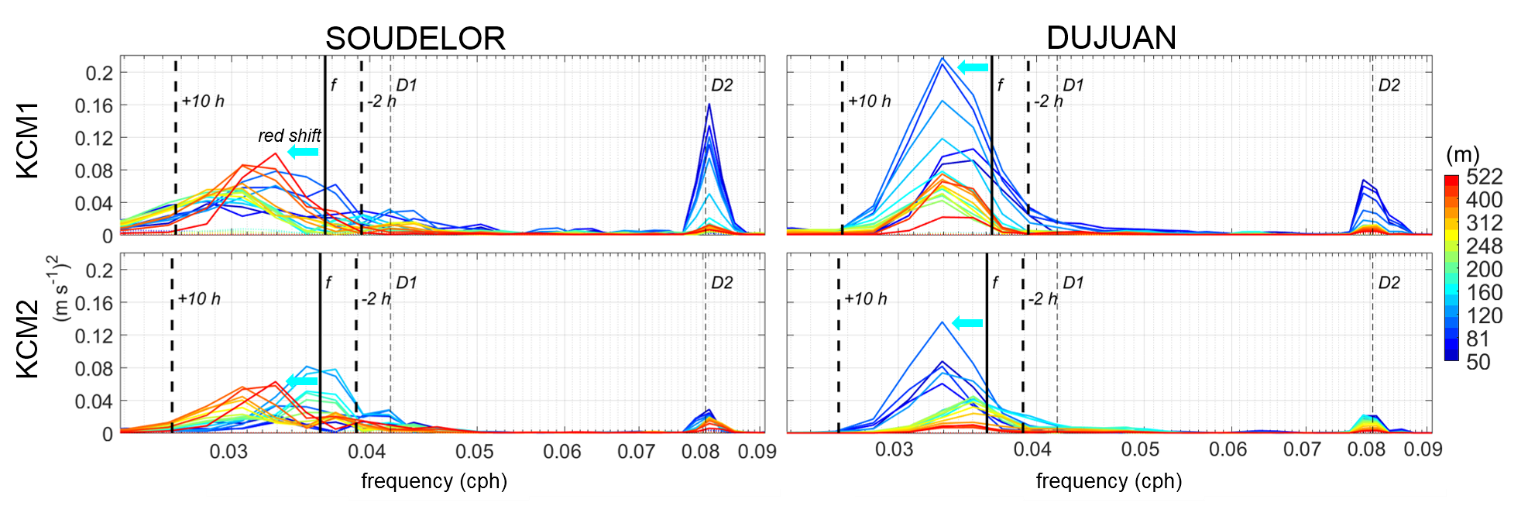


Figure S3 Variance-preserving rotary power spectra for model-derived currents during SOUDELOR and DUJUAN. Solid lines are clockwise components and dotted lines (faint and weak) are anticlockwise components. Vertical solid line is local inertial frequency (*f*) and vertical dashed lines indicate near-inertial frequency band for analysis. Vertical dotted lines mark the diurnal (D1) and semidiurnal (D2) frequencies. Colours indicate depths.

NIWs from the model outputs during typhoons CHAN-HOM and GONI are shown in S. Fig. 4. During typhoon CHAN-HOM, the simulated and observed NIWs were both weak (Fig. 1b, c). The simulation results show that NIWs induced by CHAN-HOM were preferentially located on the right-hand side of the typhoon track near the sea surface (4-m depth) (Supplementary Video 7). The simulated waves were about 0.2 m s^-1^ at KCM1 and 0.35 m s^-1^ at KCM2 at 14 h GMT on July 10^th^ when the most intense NIWs were generated around the mooring sites during CHAN-HOM passage (S. Fig. 4a). Wave amplitudes at 100-m depth were less than 0.05 m s^-1^ at the mooring sites ~3 days later (S. Fig. 4b; Supplementary Video 8), in contrast to the case of typhoon SOUDELOR (Fig. 1b, c). Depth-integrated kinetic energy greater than 3000 J m^-2^ appeared in the Kuroshio downstream, but not at the observation sites or upstream of them (S. Fig. 4c); this is a result of downstream energy propagation along the Kuroshio. At the mooring sites during typhoon GONI, which passed nearby along the right-hand side of the Kuroshio, NIWs from the observations and the simulation agreed well; they were energetic (> 0.3 m s^-1^) in the surface layer (4 m) and stronger at KCM2 than at KCM1 (Fig. 1b, c, S. Fig. 4d, Supplementary Video 9). Wave generation was weak in the Kuroshio upstream, but strong downstream. After ~3 days, NIWs at 100-m depth were weak at the mooring sites (S. Fig. 4e, Supplementary Video 10). Depth-integrated kinetic energy was prominent on the right-hand side of the Kuroshio downstream from the mooring sites (S. Fig. 4f). Because the Kuroshio advects NIWs downstream, their 100-m and depth-integrated energies were weak at the mooring sites for typhoons CHAN-HOM and GONI.


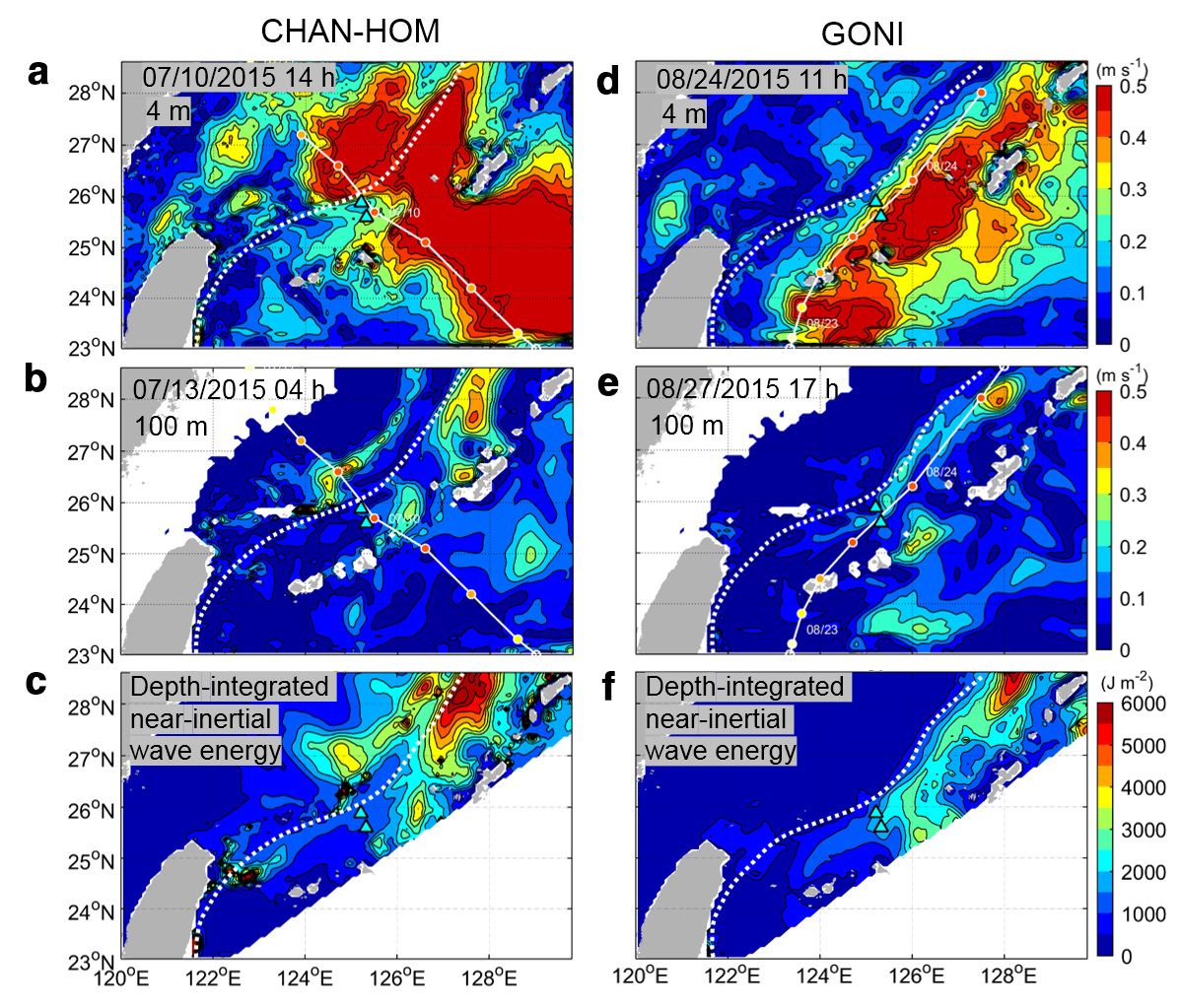


Figure S4 a, Snapshot of NIW amplitude near the sea surface (4-m depth) for typhoon CHAN-HOM on July 10^th^, 2015 (14 h GMT). b, Snapshot of NIW amplitude at 100-m depth on July 13^th^ (04 h GMT) for CHAN-HOM. c, Depth-integrated NIW kinetic energy over 20 days during typhoon CHAN-HOM. d, Snapshot of NIW amplitude near the sea surface (4-m depth) for typhoon GONI on August 24^th^, 2015 (11 h GMT). e, Snapshot of NIW amplitude at 100-m depth on August 27^th^ (17 h GMT) for GONI. f, Depth-integrated NIW kinetic energy over 20 days during typhoon GONI. Triangles mark the observation stations KCM1 and KCM2 as shown in Figure 1a.
